# Assessing New Methods to Optimally Detect Episodes of Non-metabolic Heart Rate Variability Reduction as an Indicator of Psychological Stress in Everyday Life: A Thorough Evaluation of Six Methods

**DOI:** 10.3389/fnins.2020.564123

**Published:** 2020-10-22

**Authors:** Stephen B. R. E. Brown, Jos F. Brosschot, Anke Versluis, Julian F. Thayer, Bart Verkuil

**Affiliations:** ^1^Department of Health, Medical, and Neuropsychology, Leiden University, Leiden, Netherlands; ^2^Leiden Institute for Brain and Cognition, Leiden, Netherlands; ^3^Humanities and Social Sciences, Department of Psychology, Red Deer College, Red Deer, AB, Canada; ^4^Department of Clinical Psychology, Leiden University, Leiden, Netherlands; ^5^Department of Public Health and Primary Care, Leiden University Medical Centre, Leiden, Netherlands; ^6^Department of Psychological Science, University of California, Irvine, Irvine, CA, United States

**Keywords:** additional HRV, psychological stress, worry, cardiovascular disease, heart rate variability

## Abstract

Frequent or chronic reduction in heart rate variability (HRV) is a powerful predictor of cardiovascular disease, and psychological stress has been suggested to be a co-determinant of this reduction. Recently, we evaluated various methods to measure additional HRV reduction in everyday life and to relate these reductions to psychological stress. In the current paper, we thoroughly evaluate these methods and add two new methods in both newly acquired and reanalyzed datasets. All of these methods use a subset of 24 h worth of HRV and movement data to do so: either the first 10 min of every hour, the full 24 h, a combination of 10 min from three consecutive hours, a classification of level of movement, the data from day *n* to detect episodes in day *n* + 1, or a range of activities during lab calibration. The method that used the full 24 h worth of data detected the largest percentage of episodes of reduced additional HRV that matched with self-reported stress levels, making this method the most promising, while using the first 10 min from three consecutive hours was a good runner-up.

## Introduction

With cardiovascular disease being the dominant cause of death in the world (Alwan, 2011), studying predictors of this serious ailment is imperative. The development of cardiovascular disease can be powerfully predicted by frequent or chronic reductions in the variation of time between successive heart beats (i.e., by reductions in so-called heart rate variability or HRV; Bosma et al., 1998; Orth-Gomér et al., 2000; Matthews and Gump, 2002; Rosengren et al., 2004; Kivimäki et al., 2006) as well as to precede the development of several risk factors, like hypertension, high cholesterol, diabetes, and immunological markers of pathogenic states (Thayer and Lane, 2007; Thayer et al., 2010; Jarczok et al., 2019). The risk of negative cardiovascular events may be increased by as much as 32–45% by low HRV (Hillebrand et al., 2013) and a potential cause of such HRV reductions may be prolonged exposure to psychological stress (McEwen, 2001). Stress can be considered to be a complicated, multidimensional phenomenon that may be strongly related to the consistency of a person’s emotional response to life events (e.g., with happiness, anger, etc., Lazarus, 1993).

The relationship between stress and changes in physiological parameters that are not due to changes in physical activity was demonstrated first by Blix et al. (1974) in helicopter pilots during take-off. Our lab has recently developed a technique to detect episodes of reduced HRV in ambulatory participants (Verkuil et al., 2016), and to relate these physiological episodes to the participants’ self-reported episodes of psychological stress and worrying (Verkuil et al., 2016; Brown et al., 2018). We had participants wear an ECG sensor for 24 h, as they went about their daily doings. Each experimental session started with a short calibration period in the lab, during which participants engaged in four classes of energy-expending physical activity that might also be performed during a regular day: standing, cycling, climbing stairs, and lying down. The participants’ HRV was measured during each of these classes of activity. We used these data to compute an HRV baseline during various levels of physical activity^1^. Some studies have excluded all periods of high physical activity (Brosschot et al., 2007; Pieper et al., 2007, 2010), and/or have identified epochs of non-movement by using accelerometer readings (Sowder et al., 2010), but the interrelatedness of activity levels and HRV (Rennie et al., 2003) makes it imperative to take levels of physical activity into account when studying HRV. Research has consistently shown that during episodes of physical activity, heart rate increases are associated with HRV decreases (for an extensive review, see Michael et al., 2017). A number of other factors influence HRV (reviewed in Fattison et al., 2016), and we therefore only include participants who neither smoked, nor were on antihypertensive or cardiological medication like beta-blockers (also see the Discussion). Our approach enabled us to estimate the amount of HRV that is not due purely to physical activity (i.e., *additional physiology*, in the current study, HRV: the term *additional* was coined by Blix et al., 1974) and this, in turn, allowed us to examine the relationship between psychological factors such as stress and emotions and physiological activity.

In the Verkuil et al. (2016) study, the HRV and physical activity data that were collected during a calibration period in the lab, were used to compute personalized algorithms for each participant, which could then be utilized to detect episodes associated with reductions in additional HRV. Given that these episodes of reduced additional HRV were not associated with concurrent levels of movement, they can therefore considered to be related to psychological stress (Myrtek et al., 2005). Furthermore, participants were prompted hourly to fill out some questionnaires on mobile phones while wearing their ECG sensors. The questionnaires assessed whether participants had experienced stress or worry during the previous hour, enabling us to associate physiological stress markers and psychological stress markers.

Techniques to estimate additional physiology are involved methods that have only occasionally been used in ambulatory emotion-related studies; furthermore, heart rate was typically studied instead of HRV and, crucially, no *individualized algorithms* were utilized (Myrtek and Brügner, 1996; Myrtek, 2004; Myrtek et al., 2005; Ebner-Priemer et al., 2007; Prill and Fahrenberg, 2007). Verkuil et al. (2016) developed a two-step process to detect episodes of additional HRV reductions: first, the relationship between HRV and movement (i.e., walking, cycling, etc., all expressed as acceleration) was formalized by fitting an inverse regression model to the data that were acquired during the calibration period. This was done separately for each participant. The obtained model parameters were then utilized to detect episodes of reduced additional HRV (see below, as well as Verkuil et al., 2016, for more detail). In a follow-up study, we then related these episodes of reduced additional HRV to episodes of worry or stress that were self-reported by participants (Brown et al., 2018). In the same study, we explored alternative methods to that used by Verkuil et al. (2016). All of these methods, which are detailed in the “Materials and Methods” section below, are based on a similar principle: the construction of an inverse regression model that quantifies the relationship between HRV and movement. However, these alternative methods use different subsets of the data to compute those inverse regression models. For example, instead of using data obtained during a calibration phase, as Verkuil et al. (2016) did, in one of our alternative methods, we used the first 10 min worth of data from every available hour to create our inverse regression model. One alternative method seemed particularly promising: simply using all available data (in the Verkuil et al., 2016, study, that yielded 24 h worth of data) for a given participant led to a considerably better match between physiological episodes of reduced additional HRV and self-reported psychological episodes of worry and stress than using the data from the lab calibration.

We have evaluated these alternative methods by reanalyzing data and found promising results, so further tests on a dataset that was acquired for that specific purpose was in order. We have therefore acquired data for six participants, who were subjected to three 24 h test sessions. Not only did this allow us to test our methods on a dedicated dataset, but it also allowed us to evaluate two more methods to further explore the optimal method to estimate reductions in additional HRV. First of all, we have added a class of activity to Verkuil et al. (2016) calibration phase in the lab: participants were required to read a complicated text out loud and they had to clench their fists and tense their shoulders for 3 min. We expected that reading out loud while the experimenter listened would evoke feelings of stress in the participants, while clenching their fists and tensing their shoulders might reduce their HRV, which is common for such isometric activity (i.e., clenching muscles without actually moving; Stewart et al., 2007). Incorporating this “stress-induction” class of activity into the lab calibration phase may therefore improve the sensitivity to detect episodes of reduced additional HRV in the method that used lab calibration data. Having access to 72 h worth of data for every participant allowed us to introduce and explore one further method: it might be possible to use the data of day *n* to detect episodes of reduced additional HRV in day *n* + 1. This method has the advantage of avoiding “double dipping” into the data by using an inverse regression model that was established on one dataset (i.e., one 24 h period) to detect episodes in another dataset (i.e., another 24 h period) of the same participant.

Given the modest sample size of the current study—although the results were clear and in line with earlier work—we have also reanalyzed a dataset from our lab, which also included 72 h worth of data for participants (Versluis et al., 2018). However, we were unable to test all of our hypotheses in this dataset alone, as the participants in this dataset were not subjected to the laboratory calibration phase that was used by Verkuil et al. (2016).

The goal of the current paper is to further explore the optimal way to study and quantify the relationship between additional HRV reductions and movement, to learn more about this powerful and important predictor of cardiovascular disease. We will compare the performance of the various methods in a number of ways and, crucially, we will determine which of the methods is best able to detect episodes of reduced additional HRV, as demonstrated by a high correspondence between such method-identified physiological episodes and participant-reported episodes of psychological stress and worrying. Given our previous findings, we expect the method that used all available data for a given session to perform best. The method that used the first 10 min of three consecutive hours may also perform well, based on previous findings. Our two new methods (extended lab calibration that incorporated stress induction and using the data of day *n* to detect episodes of reduced additional HRV in day *n* + 1) may, in turn, outperform these two methods.

## Materials and Methods

### Study Design

We reanalyzed one dataset (Versluis et al., 2018, institutional review board approval number 4689348773) and we acquired data from six new participants to evaluate a number of methods to optimally detect episodes of reductions in additional HRV.

### Setting

Participants for the new dataset were tested at Leiden University in 2015 and 2016. Participants were recruited via posters in the building and through Leiden University’s digital participant recruitment system. For the Versluis et al. (2018) dataset, please see the section “Materials and Methods” section in the relevant paper.

The six new participants we tested for this study were invited into the lab, were fitted with an ECG sensor, mounted on a chest strap, and were provided with an Android-based Motorola Razr cell phone which prompted them once an hour, at random moments within that hour, to fill out a set of psychological questionnaires about whether they had experienced stress and worry during the past hour, and how long these episodes had lasted (for more information, see Verkuil et al., 2016, whose procedure was followed). Participants then undertook the following types of (physical) activity, without breaks in between: (1) sitting down for 3 min while watching a relaxing video; (2) standing up for 3 min while counting in steps of two (to keep participants’ minds off of ruminating); (3) lying down for 3 min while counting in steps of two; (4) cycling on a stationary bike for 3 min; (5) sitting down, clenching one’s fists and tensing the shoulders for 3 min; (6) reading a text on the history of Leiden University out loud for 3 min, while the experimenter listened; (7) climbing four flights of stairs (63 steps). Two types of activity were added to the Verkuil et al. (2016) lab calibration: clenching one’s fists and tensing the shoulders, which was expected to reduce HRV, and reading a complicated text out loud, which was expected to evoke feelings of stress. After engaging in all of these types of activity, participants were sent home, with instructions to wear the ECG monitor for 24 h and fill in the hourly questionnaires on the smartphones.

### Participants

We tested six participants (four females, mean age 26.5 years), who were each subjected to three 24-h test sessions. Unfortunately, one 24-h dataset was not recorded by the ECG sensor due to technical reasons; this left us with five complete 72-h datasets and one dataset of 48 h. We have included the participant for whom we had 48 h worth of data in our analyses and considered one 24 h dataset to be statistically “missing data” that were not replaced. We have also reanalyzed data from Versluis et al. (2018). Due to technical reasons which have resulted in noisy data, we were only able to analyze 5 participants from the control condition of the study from Versluis et al. (2018) (all females, mean age 26 years), which obviously limits the power of the concomitant analyses; we consider this to be a set of exploratory analyses. All participants signed informed consent before being included and they were financially compensated according to Leiden University’s policy for the remuneration of participants. For both studies, participants who smoked, or who were on antihypertensive or cardiological medication (like beta-blockers) were not allowed to participate in the studies.

### Variables

In the current study, we have evaluated six alternative methods to the additional HRV detection method that was described by Verkuil et al. (2016). All of these methods used a similar approach: an inverse regression model was fitted for every individual to quantify the relationship between HRV, expressed as the root mean squares of successive differences, RMSSD^2^, and movement, which was expressed as acceleration in *g* (the averaged acceleration in three axes), according to Eq. 1.

(1) $$E\,x\,p\,e\,c\,t\,e\,d\,R\,M\,S\,S\,D_{i,j}=B\,0_{i}+\frac{B\,1_{i}}{a\,c\,c\,e\,l\,e\,r\,a\,t\,i\,o\,n_{i,j}}$$

In this inverse regression model, an expected RMSSD value for participant *i* at 30-s sampling interval *j* was computed as the sum of the value of RMSSD while no acceleration was present (i.e., the intercept, *B0*_*i*_) and the change in RMSSD that was due to acceleration (i.e., the slope, *B1*_*i*_). The standard error of the mean of RMSSD was also computed, to be used in later computations (see section “Data Sources/Measurement” below).

RMSSD was computed for 30-s intervals throughout the entire 24-h test session and was then averaged over samples that spanned different amounts of time, depending on the particular method used (see below). *g*, as used in this study, has been demonstrated to be a valid method to measure movement, especially walking, jogging, sitting, and lying down (Lugade et al., 2014). We then fitted the inverse regression model to a subset of all the data points that were available for a given participant; for example, Verkuil et al. (2016) fitted such a model to the HRV and movement data that were acquired during a laboratory calibration period while participants performed various activities. The parameters from these regression models were then utilized, for each individual participant, to predict HRV levels as a function of movement levels. Whenever actual HRV levels fell two standard errors below predicted HRV levels, and such a difference lasted at least 7.5 consecutive minutes^3^, we considered this to represent an episode of decreased additional HRV (formulae are presented in Verkuil et al., 2016). In line with Verkuil et al. (2016), if multiple such episodes were identified within a given hour, we only used the first episode that was detected within that hour in further analyses.

### Data Sources/Measurement

All 24-h ECG data and movement data were collected with an ecgMove sensor (ECGMove 3, Movisens, GmbH, Karlsruhe, Germany). Data were processed offline in Movisens Data-Analyzer version 1.12 (preprocessing and artifact rejection were described in Verkuil et al., 2016); the Data-Analyzer software uses automated algorithms to detect and remove artifacts in the data. Data were then analyzed in MATLAB^TM^ (MathWorks, Natick, Massachusetts). Analysis scripts are available from the corresponding author. The methods that were used to detect episodes of reduced additional HRV are described next.

#### Method 1: First 10 Min of Every Hour

This method, described previously in Brown et al. (2018), computed inverse regression models based on the first 10 min of every hour for which data were collected. For example, this yielded 24 separate inverse regression models if 24 h worth of data were available. Episodes of reduced additional HRV (see above) were detected for every hour, using the model parameters for that specific hour. An advantage of this method is that episodes of reduced additional HRV within a given hour were detected with a model that was based on data from that specific hour. This “double dipping” is also this method’s disadvantage: (part of) the data from hour *n* were used to detect additional HRV episodes in hour *n*. Such episodes were also looked for in the first 10 min of data, which was the time period used to construct the inverse regression model that was utilized for this detection process in the first place. Nevertheless, this appears to be a fairly minor concern: should an episode of reduced additional HRV be identified in the first 10 min of a given hour, then there seems to be no empirical reason to question whether a person actually experienced worry or stress during those 10 min (also see section “Discussion”).

#### Method 2: Full Dataset

This method used the entire period for which data were collected during a given test session to compute an inverse regression model. For example, if 24 h worth of data were available, a single inverse regression model was computed, based on that entire 24-h period. The parameters from this model were then used to identify episodes of reduced additional HRV separately for every hour in that dataset. The inverse regression models in this method are based on a large number of data points, rendering these models more robust than models based on just 10 min worth of data, which is an advantage this method offers. This advantage is slightly offset by the levels of movement and HRV being averaged over the total available time period and them not being modeled separately for each hour. This method has been described previously in Brown et al. (2018).

#### Method 3: First 10 Min of Three Consecutive Hours

As discussed previously in Brown et al. (2018), this method computed an inverse regression model based on the first 10 min worth of data for three consecutive hours, which allowed us to compensate for fluctuations over time in movement or HRV. In this method, every regression model was therefore based on 30 min worth of data. The inverse regression models for the first and last hours of a dataset were based on the first 10 min of the first and second hours of the dataset and on the first 10 min of the penultimate and final hours, respectively. So, if 24 h worth of data were available, we computed 24 separate inverse regression models. An advantage of this method is that changes in HRV or movement levels over three consecutive hours were taken into account. Furthermore, the inverse regression models were based on an average of the first 10 min of three consecutive hours, which leads to more reliable model parameter estimations than 10 using only 10 min worth of data would (cf. Method 1, above). However, using HRV and movement data from three consecutive hours is also a disadvantage: more data may be used to compute inverse regression models, but movement and HRV levels are autocorrelated over time, which would reduce variation in these levels over three consecutive hours.

#### Method 4: Movement Level Bins

This method, discussed previously in Brown et al. (2018), used the natural variation in a participant’s movement levels throughout the day. We therefore binned movement data based on quartiles that were defined per participant, thus creating four bins that classified levels of movement that ranged from relatively very low to relatively very high. Each bin contained 5 consecutive minutes’ worth of data: a participant’s level of movement within these 5 min had to be in between two quartiles to be assigned to a specific bin. Given our desire to identify all four bins for as large a number of participants as possible, we chose to base bins on periods of five consecutive minutes; making bins wider (i.e., encompassing more time), would attenuate the number of participants for whom all four bins could be identified (see below). Our analysis was restricted to the 5 min bin of every movement class that occurred first. For example, if four clusters of quartile-1 movement were identified for a given participant, only the first cluster was used in subsequent analyses, so that each bin contained the same amount of data for each participant (e.g., not 5 min for one participant and 80 min for another participant). Inverse regression models were based on all available quartile data, so a maximum of 20 min worth of data. By representing four levels of movement in the inverse regression models, this method takes variations in an individual’s movement into account, which is an advantage that is not offered by methods that only use the data from the first 10 min of every hour, which are, of course, unlikely to contain each possible level of movement by mere chance. Another advantage of this method is that it only requires the computation of a single inverse regression model, as opposed to methods that require models for every hour of data. Unfortunately, not all four bins could be identified in one of the six newly tested participants. Of course, this participant did have movement data that fell between two quartiles, but s/he simply did not have 5 consecutive minutes’ worth of such data. However, the other three bins could be identified in this participant, yielding 15 min worth of data that were usable to compute an inverse regression model. The same applied to the five reanalyzed participants from the Versluis et al. dataset. In a way, this activity bin method is comparable to the laboratory-based method of Verkuil et al. (2016), which was based on four predetermined physical activity categories: lying down, standing, cycling and climbing stairs. However, the activity bin method it is data-driven and does not depend on a laboratory-based calibration data, which might not be available to interested researchers, and this renders the activity bin method more versatile.

#### Method 5: Extended Calibration

This method is highly comparable to the original method by Verkuil et al. (2016): we computed an inverse regression model on the data acquired during the lab calibration, but we added two types of activity: reading a complicated text out loud and clenching one’s fists while tensing one’s shoulders. We expected these two activities to be stressful and to lower HRV, respectively. Therefore, adding these two types of activity may lead to the formulation of a model that has a better fit than Verkuil et al. (2016) original models, as (simulated) stress and an HRV-lowering procedure are now also included in the calibration phase. A disadvantage of this method is that it is difficult to find a text that is difficult to read for all participants; although most of our participants stumbled over such words as “string galvanometer” and the Latin names of ancient professors (e.g., Jacobus Arminius, Daniel Heinsius, etc.), other participants read through these texts with few issues.

#### Method 6: Next-Day Prediction

An important question following the work that was reported by Brown et al. (2018) was whether it might be possible to use the inverse regression model of day *n* to identify episodes of reduced additional HRV in day *n* + 1. This method was performed on a subset of all available data: we used all the data for a participant’s first testing day to detect episodes of reduced additional HRV in his or her second day, and all the data from his or her second day to detect episodes in the third day. The advantage of this method is that there is no double dipping into the data, as the model used to detect episodes of reduced additional HRV in a given day is based on data from a completely different day. The obvious disadvantage was that no episodes of reduced additional HRV could be detected in the first test session (*n*), because that would require an inverse regression model to be computed for day *n* – 1; theoretically, one could use the data from, for example, the last testing day for that purpose. Given our specific interest in the efficacy of “next-day” predictions, we have not explored that option further (cf. section “Discussion”).

#### Episodes Detected During Sleep

Occasionally, our methods identified episodes of decreased additional HRV during a participant’s sleep. If actual measured HRV is well below expected HRV levels, this can happen, but clearly, such a phenomenon cannot be due to *conscious* psychological stress during a participant’s sleep. Having said that, for methods in which the inverse regression model was calculated based on data acquired during waking periods, these episodes of decreased additional HRV may have meaning, given that an association between low sleeping HRV and preceding stress has been reported (e.g., Hall et al., 2004; Brosschot et al., 2007). This relationship has been suggested to reflect unconscious stress-related cognition (Brosschot, 2010; Brosschot et al., 2010). Given that nocturnal HRV was not the primary focus of this paper, we have chosen not to include such episodes in our analyses.

### Bias

Given that there was no experimental manipulation in this study, experimenter bias toward participants was minimized. Given that all analyses were automatized through scripts, and the outcome variables were the number of episodes of reduced additional HRV that were detected by those scripts, experimenter bias and subjectivity were minimized.

### Study Size

Following the work reported by Brown et al. (2018), we continue to explore and refine our methods to detect episodes of reduced additional HRV. Our choice of sample size reflects this exploratory nature (also see the Discussion).

### Quantitative Variables

The handling of quantitative variables was described in detail under Data sources/management.

### Statistical Methods

The current paper’s objective was to evaluate the efficacy of the six methods described above in detecting episodes of reduced additional HRV. To this end, we have compared these methods in three ways, following the strategy introduced in Brown et al. (2018). Two of these comparisons served to demonstrate the variation in number of episodes of reduced additional HRV that was identified by each method. Firstly, to explore the variation in numbers of identified episodes of reduced additional HRV, we compared the number of episodes each method detected by using repeated-measures analyses of variance (ANOVAs) with test day and the different additional HRV estimation method (e.g., first 10 min of every hour, etc.) as within-subjects factors. We then computed Pearson correlations between the numbers of episodes that were detected in three 24 h periods, to test the temporal reliability of these methods. These two analyses have been performed for both datasets analyzed here.

In our newly acquired data, participants reported once an hour whether they had been stressed or worried during the past hour. We then performed a vital analysis, in which the onsets of participants’ self-reported episodes of psychological stress and worry were compared to the onsets of the episodes of reduced additional HRV that were identified by each of the methods. The first two analyses charted the distribution of the number of physiological episodes of reduced additional HRV that the different methods identified, as well as the temporal reliability of each method, but this key comparison revealed true episodes of reduced additional HRV by demonstrating to what extent these *physiological* events matched up with *psychological* events of stress and worry. We have therefore computed, separately for every method and for every available hour worth of data, the percentage of participants that had matches between episodes of reduced additional HRV and self-reported stress and worry episodes. We then calculated the average percentage of such matches within a given method. These three comparisons are expected to assess the quality of each of the methods discussed here and to ascertain which of these methods seems to be the best alternative to the laboratory calibration method presented by Verkuil et al. (2016).

## Results

### Reanalysis of Versluis and Colleagues Data

As there was no calibration phase in the study by Versluis et al. (2018), we utilized four methods to identify episodes of additional HRV: we used the first 10 min of every hour, the full dataset of 24 h, the method that used a combination of the first 10 min of three consecutive hours, and the method that used activity type bins. Some methods identified more reduced additional HRV-episodes than others, as presented in Table 1.

**TABLE 1:** Mean number of additional HRV episodes (*SD*) identified in three test days of 24 h each.

	First 10 min of every hour	Full 24 h of first test day	First 10 min of 3 h	Activity type bins
T1	9.6 (2.6)	16.4 (2.1)	12.4 (1.1)	5.2 (3.3)
T2	7.8 (3.7)	14.6 (3.1)	12.8 (2.9)	11.0 (6.1)
T3	6.8 (1.9)	13.6 (2.0)	10.4 (1.1)	14.0 (2.5)
$\bar{X}$	8.1	14.9	11.9	10.1

*Calibration time period was also included in detection periods. Every method detected additional HRV episodes in all five analyzed participants.*

A repeated-measures ANOVA with test day and estimation method as within-subjects factors revealed a significant difference between methods, *F*(3, 12) = 25.5, *p* < 0.0005, η*_*p*_*^2^ = 0.72. This effect suggested that the method that used the full dataset identified the largest mean number of episodes of reduced additional HRV (14.9), while the methods that used the first 10 min of every hour identified the lowest number of episodes (8.1). There was no reliable effect of test day, *F*(2, 8) = 0.14, *p* = 0.87, η*_*p*_*^2^ = 0.03, but test day and method interacted, *F*(6, 24) = 10.0, *p* < 0.0005, η*_*p*_*^2^ = 0.72. Three of the four methods investigated here seem to identify relatively robust numbers of additional HRV episodes over time, but this interaction seems to be driven by the outlying observation in the first test day for the method that used activity class to detect reduced additional HRV episodes (5.2 episodes in the first session vs. 11.0 and 14.0 episodes in the second and third sessions, respectively). This may be an artifact of the low power of the analyzed sample, as there is no theoretical reason to assume this method would identify a lower number of additional HRV episodes in one of the three 24 h periods that were analyzed. Furthermore, pairwise comparisons suggested that the only significant differences in identified additional HRV episodes were those between the first and second test day for the activity class method, *t*_4_ = 3.7, *p* = 0.02 and between the first and third times series for the activity class method, *t*_4_ = 4.5, *p* = 0.01. All other differences were not significant (all *p*s > 0.06).

To further explore the reliability of the methods over time, we computed Pearson correlations between the average number of episodes detected over time, separately for every method. The largest correlation observed was the one between the number of episodes detected in the second and third test day for the method that used a combination of 10 min from three consecutive hours, *r* = −0.97, *p* = 0.008, CI_95_ = [−0.99, −0.61]. The correlation between the first and second test day for the method that used activity type was also large, *r* = 0.88, *p* = 0.051, CI_95_ = [-0.01, 0.99]. The lack of statistically robust correlations is likely due to the low power of the analyzed sample: we therefore urge the reader to interpret these correlations as effect sizes without reliance on the associated *p*-values; excepting the activity class method, all methods seem to identify numerically relatively similar numbers of additional HRV episodes over time. Note that the method that used the full dataset to identify episodes of reduced HRV, which was associated with a good match between episodes of self-reported stress and worry and method-identified episodes of additional HRV in other work (Brown et al., 2018), also appears to provide a stable estimate of additional HRV reduction episodes over time (all *r*s between 0.70 and 0.80). Unfortunately, the crucial test, in which self-reported psychological stress and worry episodes are related to method-identified physiological episodes of reduced additional HRV, could not be performed for the Versluis et al. (2018) dataset, as there was no hourly self-reported stress or worry data available for these participants. This was a powerful motivation to test additional participants.

#### New Data to Test All Methods

We tested six new participants to further evaluate all six methods and to corroborate findings reported elsewhere (Brown et al., 2018). We first compared the numbers of episodes of reduced additional HRV that were identified by every method. Given that we could only analyze two of the three days’ worth of data for the method that used the data of day *n* to detect episodes of reduced additional HRV in day *n* + 1 (because there is no data before day 1, only days 2 and 3 could be analyzed with this method), we have evaluated that method separately. We therefore first performed a repeated-measures ANOVA with test day and estimation methods as within-subjects factors, which suggested that the various methods identified different numbers of episodes of reduced additional HRV, *F*(5, 20) = 6.6, *p* = 0.001, η*_*p*_*^2^ = 0.62.

As can be seen in Table 2, Verkuil et al. (2016) lab calibration method detected the lowest number of episodes (3.6), while the method that used the full dataset identified the largest number of episodes (11.4). Interestingly, pairwise comparisons revealed no significant differences whatsoever, suggesting that the different methods each identified reliable numbers of episodes of reduced additional HRV over time. Furthermore, it is interesting to note that, once again, the method that used the full dataset identified the largest number of episodes overall. There was no reliable effect of test day on number of episodes detected, nor did method and test day reliably interact (*p*s of 0.29 and 0.49, respectively).

**TABLE 2:** Mean number of additional HRV episodes (SD) identified in three test day of 24 h each.

	Verkuil et al. lab calibration	First 10 min of every hour	Full dataset (3 × 24 h)	First 10 min of 3 h	Activity type bins	Extended lab calibration
T1	3.8 (3.1)	6.4 (2.7)	12.4 (3.9)	8.4 (3.2)	6.2 (5.5)	4.4 (4.2)
T2	3.8 (3.6)	6.2 (2.1)	11.5 (2.2)	6.7 (3.7)	5.0 (2.6)	4.5 (4.2)
T3	3.2 (1.6)	6.2 (3.2)	10.3 (2.6)	7.2 (3.7)	6.8 (4.3)	3.7 (3.2)
$\bar{X}$	*3.6*	*6.3*	*11.4*	*7.4*	*6.0*	*4.2*

*Calibration time period is also included in detection periods. Every method detected additional HRV episodes in all five analyzed participants. The bottom row contains the number of identified episodes averaged over test day.*

We then computed Pearson correlations between numbers of episodes detected over time, again, separately for every method: we have, once more, treated these correlations as effect sizes. The largest correlation was for the numbers of episodes detected in test days 1 (6.1 episodes) and 3 (6.8 episodes) for the method that used bins of activity type, *r* = 0.95, *p* = 0.01, CI_95_ = [0.61, 0.99]. The majority of correlations (11 out of 15) exceeded *r* = 0.40. Clearly, these correlations are based on a low-powered dataset. Furthermore, these low correlations are not necessarily indicative of poor temporal reliability of the methods evaluated here, as participants could simply have experienced different numbers of episodes of reduced additional HRV on the various days they were tested on.

We performed another repeated measures ANOVA like the one described above, but we now incorporated the final method, which used the data from day *n* to detect episodes of reduced additional HRV in day *n* + 1. Because this method only allowed us to analyze data from the second and third days of testing, we have only incorporated these two test days for every method analyzed. This analysis revealed a reliable difference in number of identified episodes of reduced additional HRV; even taking just the second and third test day for every participant into account, the lab calibration method by Verkuil et al. (2016) still identified the lowest number of episodes (3.5), while the method that used the data of day *n* to detect episodes in day *n* - 1 identified the largest number of episodes (11.2; 9.4 episodes during the second day and 13.0 during the third), *F*(5, 20) = 6.7, *p* < 0.0005, η*_*p*_*^2^ = 0.63. It is interesting that the latter method identified marginally more episodes than the method that used the full dataset (10.9). There was no effect of test day, nor did method and test day interact (*p*s 0.59 and 0.41, respectively).

### Associations With Worries and Stress

The crucial test for any of these methods is to see how well the physiological episodes of reduced additional HRV they detected correspond with participants’ self-reported episodes of stress and worrying. After all, the detected physiological events are only of real interest if they actually coincide with—so, represent—known episodes of worrying or stress: only then do they actually signify reductions in *additional* HRV, as opposed to possibly random physiological events in the data that may not have a clear psychological cause. Participants reported and average of 2.8 (*SD* = 3.2) episodes of worrying and/or stress.

To this end, we first calculated the percentage of participants with at least one match between an episode of method-identified reduced HRV and an episode of self-reported worrying or stress in their data. These percentages were calculated separately for every method. As presented in the first row of Table 3, certain methods were characterized by considerably higher match percentages than others.

**TABLE 3:** Matches between method-identified episodes of reduced additional HRV and self-reported episodes of stress and worry (expressed as percentages).

	Verkuil et al. lab calibration (%)	First 10 min of every hour (%)	Full dataset (3 × 24 h) (%)	First 10 min of 3 h (%)	Activity type bins (%)	Extended lab calibration (%)	Next day detection (%)
Participants with ≥ 1 match	42.9	78.6	85.7	85.7	78.6	42.9	60.0
Mean percentage of matches	50.9	65.5	70.1	73.6	59.8	53.0	68.7

*The first row presents percentages of participants with at least one match between method-identified episodes of reduced additional HRV and self-reported episodes of stress and worry. The second row presents, for this subset of participants, the percentage of average matches between method-identified episodes of reduced additional HRV and self-reported episodes of stress and worry.*

For example, the method that utilized the full dataset was associated with an 85.7% match between participants’ self-reported stress and worrying episodes and the episodes of reduced additional HRV identified by this method; this method was also found to be very promising in other work (Brown et al., 2018). Interestingly, the method that used the first 10 min of three consecutive hours to detect episodes of reduced additional HRV was associated with an identical percentage of matches. The Verkuil et al. (2016) lab calibration method was associated with a match of 42.9%. This would suggest that the methods that used all available data for a given test session and that used a combination of 10 min of three consecutive hours’ worth of data are more sensitive in detecting actual episodes of reduced additional HRV than Verkuil et al. (2016) lab calibration method, which we considered to be a golden standard. Indeed, earlier work (Brown et al., 2018) had identified the method that used a combination of 10 min of three consecutive hours to be a good “runner-up” to the method that used the full dataset.

The percentages reported above merely indicate how many participants had at least one match between method-detected episodes of reduced additional HRV and self-reported stress or worry episodes. A crucial next step is to compute the percentage of matches between method-detected episodes of reduced additional HRV and self-reported stress or worry episodes *within* the participants with at least one such match. Those percentages reveal how well, on average, each method was able to identify physiological episodes of reduced additional HRV that match and therefore represent self-reported episodes of stress or worry (provided that a participant had at least one such match). These percentages are listed in the second row of Table 3. Once more, the methods that used the full dataset and a combination of 10 min of three consecutive hours appear the most promising, with an average match between method-identified episodes of reduced additional HRV and self-reported stress worry of 70.1 and 73.6%, respectively. Although the method that used the data of day *n* to identify episodes of additional HRV reduction in day *n* + 1 performed relatively well, with a matching percentage of 68.7%, this method was only able to detect matches in 60.0% of all tested participants. The extended calibration method performed similarly to the Verkuil et al. (2016) lab calibration method, which is not surprising, given that it is based on that method. Taken together, all of the results above suggest that using the full dataset to create an inverse regression model, which will then be used to detect episodes of reduced additional HRV, or using a combination of the first 10 min of three consecutive hours’ worth of data to create such an inverse regression model appear to be the most promising methods. These two methods were associated with the best overall match between additional HRV physiology and self-reported stress and worry in the highest number of participants. Interestingly, all of the methods evaluated here identified more physiological reduced additional HRV episodes than that participants reported episodes of stress or worrying. The methods identified more episodes, on average, than participants reported in 85.2% of cases.

## Discussion

The goal of the current paper was to evaluate several methods that can be used to identify episodes of reduced additional HRV and to identify the optimal method. As in previous work (Brown et al., 2018), we found that using every data point available in a given dataset appears to be a very promising method. Interestingly, the method that was identified to be a “runner-up” in our earlier study, slightly outperformed the method that used the full dataset in the current experiment: using the first 10 min of three consecutive hours to detect episodes of reduced additional HRV also led to a very good match between such physiological episodes and self-reported episodes of psychological stress and worry. Finally, the method that used the first 10 min of every available hour to detect episodes of reduced additional HRV also seemed to perform well enough to be considered an interesting option. The advantage of all three of these methods is that they do not rely on a calibration phase in the laboratory, which facilitates the identification of episodes of reduced additional HRV for researchers who do not have access to the calibration procedure introduced by Verkuil et al. (2016).

Two new methods were evaluated here: introducing an additional class of activity during the calibration phase, and using the data from day *n* to detect episodes of reduced additional HRV in day *n* + 1. These two methods did not perform as well as the three methods described above: the extended calibration method performed similarly to the Verkuil et al. (2016) calibration method; the method that used data from day *n* to detect episodes in day *n* + 1 seemed the more promising of the two new methods, and it may therefore warrant further exploration. One problem with this method is that it requires the acquisition of at least two sessions’ worth of data, while episodes of reduced additional HRV are detected in only one of those sessions. Of course, one could devise ways to utilize the data of the “lost” session, for example by using the data of the last available test session to create an inverse regression model and to then use the resulting model parameters to detect episodes of reduced additional HRV in the first available test session. Clearly, this would complicate the method, as one would no longer be using a predictive model to detect episodes, but one would be using a kind of retroactive “prediction.” For this reason, and given the method’s overall unpromising level of performance, we have not explored this method beyond simply analyzing the data from the second and third test sessions in this paper.

One issue that we have commented on before (Brown et al., 2018), but that also characterizes the data of the six participants we have tested here, is the discrepancy in numbers of self-reported episodes of psychological worry and stress and method-identified physiological episodes of reduced additional HRV. The numerous methods invariably identify (occasionally, considerably) larger numbers of episodes of reduced additional HRV than that participants report episodes of stress and worry. For example, on average, the method that used the full dataset identified 11.4 episodes of reduced additional HRV, averaged over three test sessions, while participants reported 2.8 episodes of psychological stress or worry, averaged over three test sessions. It seems implausible that this effect is caused by technological glitches in the cell phones that were used to record participants’ responses to questionnaires about their current psychological state. This leaves two explanations: either participants underreported episodes of stress and worry for any number of reasons (social expectations, shame, forgetfulness, etc.), or the episodes of reduced additional HRV that were detected by the various methods but that were not matched by self-reported stress and worrying episodes were due, at least in part, to unconscious stress. The latter option is corroborated by other work from our lab (Brosschot, 2010; Brosschot et al., 2010).

Another explanation could be that positive psychosocial events evoked these yet unexplained reductions in HRV. Indeed, previous work has shown that such events can also lead to increases in heart rate and therefore to concomitant reductions in HRV (see, e.g., Jacob et al., 1999). However, *sustained* increases in heart rate have been shown to be associated with events of negative valence only (Brosschot and Thayer, 2003). Given that the duration of our additional HRV reduction detection episodes was 7.5 min, and given that Brosschot and Thayer showed that heart rate already starts to decrease (and therefore, HRV starts to increase) after 5 min of being presented with a positively-valenced stimulus, we do not believe that additional HRV reductions due to positive psychosocial events provide an alternative explanation of our findings.

We also acknowledge that there are many factors that are known to affect HRV (Fattison et al., 2016; Sammito and Böckelmann, 2016), such as various cardiopulmonary diseases and metabolic diseases like diabetes mellitus, as well as lifestyle habits like alcohol consumption and smoking, plus external factors like heat. Of course, even respiration itself affects HRV. It is impossible to control for every possible confound in a study, but we believe that by excluding smokers and participants who used antihypertensive or cardiological medications, we have excluded some major confounds. Our participants were also relatively young students who typically were in good physical shape. Generally speaking, it is difficult to study the relationship between HRV and metabolic demands, because the increased heart rate that accompanies intense physical exercise complicates the delineation of the different non-neural mechanisms such as respiration, which are thought to underlie HRV changes under such intense circumstances (Casadei et al., 1995, 1996; Cottin et al., 2004, 2006; Michael et al., 2017). Furthermore, chronic anxiety is associated with increased autonomic tone (for a review, see Curtis and O’Keefe, 2002), and adverse childhood events can also affect HRV (Aimie-Salleh et al., 2018; Bakema et al., 2020). This may lead to baseline differences in HRV, but the current study was not designed to address such factors. Generally speaking, we believe that additional HRV decreases as measured with our methods can be interpreted to reflect psychosocial stress; of course, there might be other, unmeasured, sources of stress as well as long-term physiological baseline differences that our methods cannot currently detect. Having said that, now that our methods to estimate reductions in additional HRV have been evaluated twice, it would be very interesting to use them to further explore the role of these kinds of factors that are known to affect HRV. In future work, we intend to combine the methods described in the current paper with between-subjects designs, which will allow us to assess baseline differences in HRV, as well as potential underlying causes of such differences.

Currently, there is debate about whether or not methods that estimate HRV, like the RMSSD method that we used in this and previous papers, should be corrected for heart rate (for a review, see de Geus et al., 2018). This is an important matter, because as the interval between two heart beats increases, so does the variability of this interval (de Geus et al.), making heart rate a potential confound. We have chosen not to correct HRV for heart rate because there is strong empirical evidence to suggest that such corrections are not necessary (e.g., Thayer et al., 2020) or even do more harm than good, by removing variance that is due to autonomic or neuropsychological processes (de Geus et al., 2018).

Our findings have clear clinical relevance: episodes of reduced additional HRV cannot be sensed by people, but they do represent periods of either psychological or physical health (or both, of course). Therefore, methods to optimally and objectively detect such episodes can be of great use to clinicians as well as to end users both by signaling epochs during which interventions are most desirable (or even required) and by teaching end users what triggers evoke such epochs. One challenge for future work will thus be to further develop ways to probe participants’ reduced additional HRV periods, and to assess all possible causes of these reductions. If these are indeed strongly related to bouts of (mindless) stress and worry, probing end users (e.g., patients suffering from stress-related (psychological or somatic) pathology) on their smartphones might become a new strategy to promote learning about and dealing with stress. One could easily not only make people more aware of their physiological stress level, but also provide them with a range of interventions that can be applied in order to directly change the stress level (i.e., breathing exercises, cognitive techniques).

We are aware that the analyses reported here have been performed on relatively low numbers of participants. However, given that we have now demonstrated, in three separate datasets, that using every available data point to detect episodes of reduced additional HRV appears to be a very accurate method to do so, and given the relative computational straightforwardness of that method, we feel confident that this method is promising and will lead to accurate results. It is particularly interesting that this method requires no special calibration phase: every researcher who has HRV and movement data will be able to analyze that data using this method. Hopefully, further applications of this method by researchers in this field will further corroborate the validity of this method. We hope that this new method will lead to fruitful insights into reductions in additional HRV which, in turn, may lead to a better understanding of this powerful predictor of cardiovascular disease.

## Data Availability Statement

The raw data supporting the conclusions of this article will be made available by the authors, without undue reservation, to any qualified researcher.

## Ethics Statement

The studies involving human participants were reviewed and approved by the Leiden University Ethics Committee. The patients/participants provided their written informed consent to participate in this study.

## Author Contributions

SB tested participants and created analysis scripts. AV tested participants. All authors contributed to the article and approved the submitted version.

## Conflict of Interest

The authors declare that the research was conducted in the absence of any commercial or financial relationships that could be construed as a potential conflict of interest.

## Footnotes

1 By physical activity, we mean bodily movement of various intensities (e.g., casual walking vs. climbing stairs).

2 There are multiple methods to estimate HRV either in the time or frequency domains. We selected this specific method because it is commonly used (Task Force of The European Society of Cardiology and The North American Society of Pacing, and Electrophysiology, 1996; Berntsen et al., 2017, p. 196) and recently, single RMSSD values were shown to be powerful predictors of health risk factors in a very large sample (Jarczok et al., 2019).

3 The Verkuil et al. method was developed in a first attempt to identify episodes of reduced additional HRV in an ambulatory setting, and it has guided the current work. Requiring actual HRV to be two standard errors below the predicted HRV for 7.5 min is a carefully considered but potentially seemingly arbitrary decision. Verkuil et al. reasoned that two standard errors would provide a pronounced enough difference between the two variables, and that 7.5 min would be enough time to qualify a period as representing worrying or stress. For a discussion of these decisions, see Brown et al. (2018).
